# Review on comparative efficacy of bevacizumab, panitumumab and cetuximab antibody therapy with combination of FOLFOX-4 in *KRAS*-mutated colorectal cancer patients

**DOI:** 10.18632/oncotarget.22471

**Published:** 2017-11-16

**Authors:** Surajit Pathak, Sushmitha S, Antara Banerjee, Francesco Marotta, Madhumala Gopinath, Ramachandran Murugesan, Hong Zhang, Bhavani B, Agnishwar Girigoswami, Jose Sollano, Xiao-Feng Sun

**Affiliations:** ^1^ Faculty of Allied Health Sciences, Chettinad Hospital and Research Institute (CHRI), Chettinad Academy of Research and Education (CARE), Kelambakkam, Chennai, India; ^2^ ReGenera Research Group for Aging-Intervention, Milano, Italy and San Babila Clinic, Healthy Aging Unit by Genomics and Biotechnology, Milano, Italy; ^3^ School of Medicine, Orebro University, Örebro, Sweden; ^4^ Gastroenterology Department, University of Santo Tomas, Manila, The Philippines; ^5^ Department of Oncology and Department of Clinical and Experimental Medicine, Linköping University, Linköping, Sweden

**Keywords:** colorectal cancer, KRAS, bevacizumab, panitumumab, cetuximab

## Abstract

Colorectal cancer, fourth leading form of cancer worldwide and is increasing in alarming rate in the developing countries. Treating colorectal cancer has become a big challenge worldwide and several antibody therapies such as bevacizumab, panitumumab and cetuximab are being used with limited success. Moreover, mutation in *KRAS* gene which is linked with the colorectal cancer initiation and progression further interferes with the antibody therapies. Considering median progression free survival and overall survival in account, this review focuses to identify the most efficient antibody therapy in combination with chemotherapy (FOLFOX-4) in *KRAS* mutated colorectal cancer patients. The bevacizumab plus FOLFOX-4 therapy shows about 9.3 months and 8.7 months of progression free survival for *KRAS* wild and mutant type, respectively. The overall survival is about 34.8 months for wild type whereas for the mutant it is inconclusive for the same therapy. In comparison, panitumumab results in better progression-free survival which is about (9.6 months) and overall survival is about (23.9 months) for the wild type *KRAS* and the overall survival is about 15.5 months for the mutant *KRAS*. Cetuximab plus FOLFOX-4 therapy shows about 7.7 months and 5.5 months of progression-free survival for wild type *KRAS* and mutant type, respectively. Thus, panitumumab shows significant improvement in overall survival rate for wild type *KRAS*, validating as a cost effective therapeutic for colorectal cancer therapy. This review depicts that panitumumab along with FOLFOX-4 has a higher response in colorectal cancer patients than the either of the two monoclonal antibodies plus FOLFOX-4.

## INTRODUCTION

Carcinogenesis is a process containing numerous steps that arise from the combination of mutations in oncogenes or tumor suppressor genes or epigenetic changes in DNA such as methylation [1]. An epigenetic factor such as abnormal DNA methylation of tumor suppressor/promoter plays a major part in the evolution of colorectal cancer (CRC) [2]. CRC is considered to be the most significant cause of cancer death worldwide. In migrant populations, it has been demonstrated that populations shifting from low- to high-risk countries are more prone to an increased cancer risk, suggesting that exposure to confined environment may have the capacity to have an effect on CRC [1]. Globally, CRC is one of the most prevalent types of cancers in developed countries. Frequency rate of CRC varies widely in different geographical areas, with fewer occurrences in Asia, Africa and parts of Latin America, but with high occurrence in Northern Europe, Australia, New Zealand and U.S. Epidemiological studies show specific components such as dietary fat and red meat to be risk factors in CRC pathogenesis [1]. Even though surgery remains the therapeutic modality for CRC, radiotherapy has shown a survival advantage over surgery with debatable reasons [3]. These outcomes further raised the question on specific application of RT and chemotherapy. Now, the main aim for clinicians is to search a predictive indicator in order to identify patients who are best suited for different therapy combination that thereby might increase the overall survival. Generally, potential predictive biomarkers include expression of oncogenes and tumor suppressor genes, markers of proliferation, angiogenesis, inflammation as well as regulation of genes involved in modulating the response to radiotherapy and chemotherapy. Of all the genes distinguished till date, operative oncogene Kirsten-ras (*KRAS*) (Kirsten Rat Sarcoma Viral Oncogene Homolog) and inoperative tumor suppressor genes like p53 and APC are found to be particularly important determinants of tumor incorporation and progression. *KRAS* gene is detected on the short arm of chromosome 12; encoding a 21kD protein required in G protein mediated signal transduction. It has constitutive GTPase activity, which gets neglected when the gene is mutated. *KRAS* mutations will promote increased and uncontrolled cellular proliferation, and malignant transformation [1]. The *KRAS* protein is turned out to be operative transiently as a response to extracellular signals such as cytokines, growth factors and hormones that trigger cell surface receptors [4]. One of the most important targets is the epidermal growth factor receptor (EGFR) which is found to be activated in colorectal carcinogenesis by the binding of ligand to its outer surface [5]. The ligand binding to the outer part of EGFR results phosphorylation of tyrosine kinase domain situated in its inner part. Then, the receptor gets activated by promoting the activation of intracellular effectors involved in intracellular signaling pathways [5]. Following the identification of two anti-epidermal growth factor receptor (EGFR)-targeted antibodies, cetuximab (Erbitux) and panitumumab (Vectibix), the treatment of CRC has stepped into the world of personalized therapies. Bevacizumab (Avastin) is another monoclonal antibody that hinders vascular endothelial growth factor-A (VEGF-A) which has been reported to be involved in certain metastatic cancers [6]. Out of these three antibodies, cetuximab is a human-mouse chimeric IgG_1_ monoclonal antibody which was approved as a second-line therapy for CRC by Food and Drug Administration (FDA) in 2004. Panitumumab is a human IgG_2_ (Immunoglobulin G_2_) monoclonal antibody which was approved as a third-line drug in 2007 by FDA [7]. The recombinant humanized monoclonal antibody, bevacizumab was approved in 2004 to combine with standard chemotherapy for metastatic CRC (mCRC). All the three antibody therapies are being used for the treatment of CRC considering the limitation of individual therapeutics. A comparative study is needed to highlight the most effective therapy among the different therapeutics. Considering the importance of *KRAS* gene mutation in CRC, an attempt is made in this review to highlight survival beneficial therapy for the CRC patients and the corresponding mostly cost effective.

### Inclusion and exclusion criteria

Studies were eligible for inclusion if they reported data from already existing databases, cross-sectional studies, case series, case-control studies, or studies with a historical control or a cohort design. Studies were desirable for inclusion if they reported on a series of patients who underwent antibody therapy alone and the combination of antibody with chemotherapy, as well as progression free survival and overall survival parameters were analyzed in the sections of “material and methods” and “results”. All studies eligible for inclusion in this review reported detailed information on the methods used to assess progression free survival (PFS) as well as overall survival (OS) parameters. When studies reported (partially) similar patient data, only the most recent and complete data sets were considered.

### Search strategy

Medline database used in our study (January 2000 to December 2016) was searched with the help of a clinical librarian. The keywords and medical subject headings (MeSH) used were “colorectal cancer”, “*KRAS* mutation”, “antibody therapy” and “FOLFOX-4” as indicated in Table 1. Only the clinical studies reported in English were selected. A manual cross-reference search of the desirable papers was performed to find additional relevant articles. Based on the primary search results, the researchers independently selected the studies that matched the inclusion criteria. Data recited as unpublished and data from the abstracts were not utilized. Any disagreements between the researchers with respect to the studies which should be included were rectified through discussion.

**Table 1:** Keywords and MeSH terms

MeSH and free text words
(“FOLFOX4 protocol”, OR “Folfox regimen”, OR “FOLFOX-4 protocol”, OR “Folinic Acid-SF”, OR “Folinic Acid SF”, OR “Leukovorin”, OR “Leukovorum”, OR “Folinic Acid”, OR “Acid, Folinic”, OR “Leucovorin, (DL)-Isomer”, OR “Calcium Leucovorin”, OR “Leucovorin, Calcium”, OR “Calcium Folinate” OR “5FU”, OR “5-FU”, OR “5-Fluorouracil”, OR “5 Fluorouracil”, OR “Fluoruracil”, OR “5-FU Lederle” OR “5-FU medac”, OR “5 FU medac” OR “Adrucil”, OR “Flurodex”, OR “Oxaliplatin) AND (“K-ras mutation”) AND (“Antibodies, Monoclonal, Humanized”, OR “Panitumumab”, OR “Cetuximab”, Or “Bevacizumab”) AND (“Colorectal Neoplasms”, OR “Colorectal cancer”).

### Data extraction

Data were extracted only from original articles using a preformatted sheet with a set of predefined parameters: type of cancer and mutation, number of patients, drug administered, drug dosage, use period, patient PFS and OS.

### Statistics

A statistician was consulted to evaluate the accuracy of our analysis. RevMan 5 was used to process the data and perform the analysis. Meta-analysis of the progression free survival and overall survival was attempted for studies presenting PFS and OS results obtained from patient’s undergone with antibody therapy alone as well as antibody therapy in combination with chemotherapy. The results were presented as weighted mean differences [95% confidence interval (CI)]. *P < 0.05* was considered to indicate that the results were significant.

## RESULTS

### Study selection, characteristics of the studies

**Figure 1:**
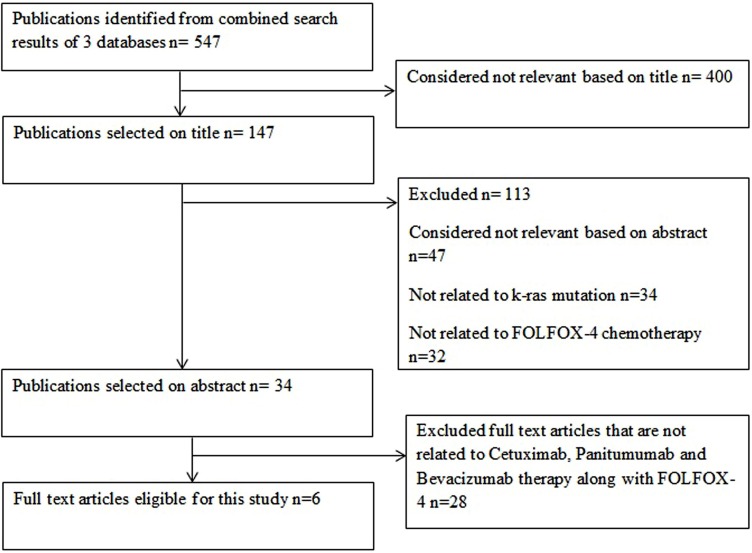
PRISMA flowchart for article screening (example)

The selection of studies according to the PRISMA flow diagram is outlined in Figure 1. Six full text articles published during 2000–2016 were screened for the review and meta-analysis among which all the studies had more than 10 patients. The details of the articles are listed in Table 2.

**Table 2:** Characteristics of the studies

1st author	Country	Total patients >10 yes =1 no= 0	Study duration	Number of patients included in the study	Patients data represented in final analysis of the study	Patients lost at follow up	Healthy controls	Diseased controls	Age: mean or median (range)	
									**WT KRAS**	**MT KRAS**
Amado et al. [9] 2008	USA	1	NM	463	427	NM	No	No	PAN 62.5(29–82)^#^ BSC 63(32–81)^#^	PAN 62(27–79)^#^ BSC 62(27–83)^#^
Douillard et al. [10] **2010**	France	1	2006–2008	1183	1096	NM	No	No	FOLFOX-4 61(24–82)^#^	FOLFOX-4 61(27–82)^#^
									PAN+FOLFOX-4 62(27–85)^#^	PAN+FOLFOX-4 63(33–83)^#^
Douillard et al. [11] **2013**	France	1	NM	1183	1060	NM	No	No	NM	NM
Karapetis et al. [13] **2008**	Australia	1	2003–2005	572	394	NM	No	No	CTX 63.5(28.6–85.9)^#^	CTX 62(37.4–88.1)^#^
Bokemeyer et al. [14] 2009	Germany	1	2005–2006	344	337	NM	No	No	FOLFOX-4 59(36–82)^#^	FOLFOX-4 61(30–75)^#^
									CTX+FOLFOX-4 59(24–74)^#^	CTX+FOLFOX-4 60(41–82)^#^
Bencsikova et al. [17] 2015	Europe	1	2005–2013	1622	1622	NM	No	No	BEV 61(22–85)^#^	BEV 63(22–83)^#^
Sharma et al. [12] 2010	USA	1	2008–2009	191	181	NM	No	No	NM	NM

Mean *=* ^*^ Median *=* ^#^ WT- Wild type; MT- Mutant; PAN- Panitumumab; BSC-Best Supportive Care ; CTX- Cetuximab; NM- Not mentioned; CRC- Colorectal cancer; Bev-Bevacizumab Disease type: CRC.

### *KRAS* mutation and antibody therapy for CRC

*KRAS*, as a proto-oncogene, is found to be mutated rapidly in CRC and has been linked with incorporation and progression of CRC [8]. In 2008, Amado *et al.* [9] evaluated the anticipative role of *KRAS* in the randomized experiment comparing panitumumab mono therapy with best supportive care and also determined whether the effect of panitumumab mono therapy on PFS differed between patients with wild (*WT*) and mutant (*MT*) type of *KRAS*. Douillard *et al.* [10] (2010) compared panitumumab-FOLFOX-4 therapy with FOLFOX-4 alone in *WT KRAS* and *MT KRAS*. Again in 2013, comparison of panitumumab-FOLFOX-4 therapy with *WT* versus *MT KRAS* was performed [11]. Sharma *et al.* [12] (2010) assessed the response and survival in mCRC patients after treating FOLFOX-4 individually and combined with bevacizumab. In 2008, Karapetis *et al.* [13] analysed tumor samples, obtained from 394 out of 572 patients with CRC who were randomly allocated to receive cetuximab. Bokemeyer *et al.* [14–15] (2009) evaluated whether the best overall response rate (ORR) of cetuximab FOLFOX-4 therapy was superior to that of FOLFOX-4 alone as first line treatment for mCRC. It has been reported that FOLFOX-4 was associated with better response rate, improved median survival and longer time for progression [16]. A recent study was done by Bencsikova *et al.* [17] (2015) on 1622 mCRC patients treated with bevacizumab along with oxaliplatin or irinotecan-based chemotherapy to monitor corresponding treatment outcomes with *KRAS* mutation status. Likewise, several randomized studies have been attempted to identify the efficacy of antibody therapy that adds up more benefits in CRC patients in combination with chemotherapy. Detail of medications administered and period of use are tabulated in Table 3.

**Table 3:** Type of medications, dose administered for the patients.

1st author	BSC (No. of patients)	FOLFOX-4 (No. of patients)	CTX + BSC (No. of patients)	CTX + FOLFOX-4 (No. of patients)	PAN + FOLFOX-4 (No. of patients)	BEV+ FOLFOX-4 (No.of patients)	FOLFOX-4 dose	CTX + FOLFOX-4 dose	PAN + FOLFOX-4 dose	BEV+ FOLFOX-4 dose
**Amado et al.****[9]** 2008	**WT K-RAS** 119	-	-	-	-		-	-	-	-
	**MT K-RAS** 100									
**Doulliard et al.****[10]** 2010	-	**WT K-RAS** 331	-	-	**WT K-RAS** 325		**WT K-RAS** OX-865 mg/m^2^ FU- 8618 mg/m^2^ FU continuous infusion 13,229 mg/m^2^	-	**WT K-RAS** PAN-62 mg/kg OX-859 mg/m^2^ LV-200 mg/m^2^ FU: 8627 mg/m^2^; continuous infusion 13,484 mg/m^2^	-
		**MT K-RAS** 219			**MT K-RAS** 221		**MT K-RAS** OX 856 mg/m^2^ FU- 8711 mg/m2 FU continuous 13,109 mg/m^2^		**MT K-RAS** PAN-57mg/kg OX-824 mg/m^2^ FU- 8294 mg/m^2^ FU continuous infusion 12878 mg/m^2^	
**Karapetis et al.** **[13]** 2008	285/572	-	287/572	-	-		-	-	-	-
**Bokemeyer et al.** **[14]** 2009	-	**WT K-RAS** 73/134	-	**WT K-RAS** 61/134	-		OX- 85 mg/m^2^ LV- 200 mg/m^2^ FU- 1000 mg/m^2^	CTX- 650 mg/m^2^ OX- 85 mg/m^2^ LV -200mg/m^2^ FU-1000 mg/m^2^	-	-
		**MT K-RAS** 47/99		**MT K-RAS** 52/99						
**Sharma et al.** **[12]** 2010	-	**83/181**	-	-	-	**WT K-RAS** 44/53	OX- 85mg/m^2^ LV- 400mg/m^2^ FU- 400mg/m^2^	-	-	BEV- 5mg/kg OX- 85mg/m^2^ LV- 400mg/m^2^ FU- 400mg/m^2^
						MT K-RAS 24/30				

BSC-Best Supportive Care; WT-Wild type; MT-Mutant; OX-Oxaliplatin; LV-Leucovorin; FU-Fluorouracil; PAN-Panitumumab; CTX-Cetuximab; - No; NM-Not Mentioned

### Effect of bevacizumab-FOLFOX-4 combination therapy on OS and PFS in CRC patients

Sharma *et al.* [12] in 2010evaluated patients with mCRC treated with first-line FOLFOX-4 with or without bevacizumab. Out of 181 mCRC patients, 83 received first-line FOLFOX-4 with or without bevacizumab and were evaluated for response. Among these patients, 44 out of 53 and 24 out of 30 received bevacizumab in combination with FOLFOX-4 in the *WT KRAS* and *MT KRAS*, respectively. In this study, no advantages were reported in the case of *MT KRAS* in terms of response or progression free survival with FOLFOX-4 based chemotherapy. Bencsikova *et al.* [17] (2015) reported a significant study on 1622 patients with mCRC who were treated with bevacizumab plus oxaliplatin or irinotecan based chemotherapy. This study suggested that *MT KRAS* does not interfere with clinical benefit from first-line treatment with bevacizumab plus chemotherapy in mCRC patients. In this study, the overall survival was found to be improved in patients treated with the first line of bevacizumab with oxaliplatin as compared to irinotecan-based chemotherapy. Patients presenting with synchronous metastases had shorter OS, and subgroup analysis showed the significance of this effect was limited to *WT KRAS* subgroup. Similar to PFS, presence of multiple metastatic sites was the risk factor for shorter OS. *KRAS* subgroup analysis of clinical outcome in the context of chemotherapy back bone confirmed similar PFS in patients treated with bevacizumab/oxaliplatin-based or bevacizumab/irinotecan based with or without *KRAS* mutation. For patients with *WT KRAS* tumors, median OS was found to be 31.0 months in bevacizumab/oxaliplatin-based subgroup and about 29.2 months in case of bevacizumab/irinotecan-based first line treatment. Whereas in patients with *KRAS* mutation, median OS was found to be 29.1 months for bevacizumab/oxaliplatin-based treatment and about 24.2 months for bevacizumab/irinotecan-based subgroup. Thus, it improved OS in patients who were started with bevacizumab plus XELOX or FOLFOX-4 which may be explained by shorter OS in *MT KRAS* patients. Sharma *et al.* [12] (2010) attempted to distinguish the PFS of *WT KRAS* and *KRAS* mCRC patients treated with first-line FOLFOX-4 (with or without bevacizumab) chemotherapy. The best ORR was 56.6% in *WT KRAS* and 50% in *MT KRAS* patients. The median PFS was about 9.3 months in *WT KRAS* and 8.7 months in *MT KRAS* populations. Median OS rate was found to be 34.8 months in *WTKRAS* was not achieved in mCRC patients. Bevacizumab remains to be investigational yet and these data do not support any predictive role for *KRAS* metastasis in response to FOLFOX-4 first-line chemotherapy.

### Effect of panitumumab and FOLFOX-4 therapy on OS and PFS in CRC patients

Previous study by Amado *et al.* [9] (2008) in CRC with the help of antibody therapies and chemotherapy described the predictive part of *KRAS* in the randomized experiment comparing panitumumab mono therapy with best supportive care (BSC). Out of 463 patients originally enrolled, 231 patients are randomly allocated to panitumumab and BSC and 232 patients were randomly allocated to BSC. Among the 231 patients, 84 of them had *MT KRAS* and 124 of them had *WT KRAS*. In the other group, out of the 232 patients, 100 of them were found to have *MT KRAS* and 119 of them were found to have *WT KRAS*. It was found that patients with *WT KRAS* had better PFS compared to patients with *MT KRAS*. Douillard *et al.* [10] in 2010 compared panitumumab-FOLFOX-4 therapy with FOLFOX-4 therapy in *WT* and *MT KRAS*. Of the total 1183 patients, 593 (∼50%) were randomly allocated to receive panitumumab-FOLFOX-4 and 590 (50%) were allocated to receive FOLFOX-4 alone. This study revealed that in the *WT KRAS*, panitumumab-FOLFOX-4 significantly improved PFS compared with FOLFOX-4 alone. In 2013, another study reported comparison of panitumumab-FOLFOX-4 therapy with *WT* and *MT KRAS*, where out of 1060 patients, 512 (48%) were identified to have *WT KRAS* and 548 (52%) were identified to have *MT KRAS* [11]. These patients were subjected to receive panitumumab plus FOLFOX-4 and inferred that improvements in overall survival rate was observed in *WT KRAS*. With *KRAS* mutation, there was a lack of response for panitumumab-FOLFOX-4 therapy [11]. In 2010, Douillard *et al.* [10] observed in *WT KRAS* patients, the median PFS was about 9.6 months for panitumumab along with FOLFOX-4 and for OS it was about 23.9 months. The median progression free survival for FOLFOX-4 alone was found to be 8.0 months and the overall survival was about 19.7 months. In *MT KRAS*, the median OS was about 15.5 months for panitumumab-FOLFOX-4 combination and about 19.3 months for FOLFOX-4 alone. Douillard *et al.* [11] again in 2013 performed a study on 512 patients without *KRAS* mutations in which PFS was found to be 10.1 months for panitumumab-FOLFOX-4 combination and 7.9 months for FOLFOX-4 alone. The OS was about 26.0 months in the panitumumab-FOLFOX-4 group whereas it was about 20.2 months for group treated with FOLFOX-4 alone. A total of 108 patients with *WT KRAS* exon 2 had other RAS mutations and these mutations were linked with inferior PFS and OS with panitumumab-FOLFOX-4 therapy, which was consistent with the findings in patients with *KRAS* mutations in exon 2.

### Effect of cetuximab and FOLFOX-4 therapy on OS and PFS in CRC patients

Karapetis *et al.* [13] (2008) analysed tumor samples, obtained from 394 out of 572 patients with CRC who were randomly allocated to receive cetuximab and it was identified that *MT KRAS* did not benefit from cetuximab, whereas *WT KRAS* got benefit from cetuximab. Bokemeyer *et al.* [14–15] in 2009 estimated whether the best ORR of cetuximab combined with FOLFOX-4 was superior to that of FOLFOX-4 alone as first line therapy with mCRC. In this study, 169 patients received cetuximab plus-FOLFOX-4 combination and 168 patients received FOLFOX-4 alone. MT *KRAS* was found in 233 out of 337 patients, 113 and 120 of them had received cetuximab plus FOLFOX-4 and FOLFOX-4 alone, respectively. This study demonstrated that addition of cetuximab to FOLFOX-4 increased the ORR compared with FOLFOX-4 alone.

Bokemeyer *et al.* [14–15] (2009) confirmed the ORR of cetuximab plus-FOLFOX-4 combination which was superior to that of FOLFOX-4 alone. Cetuximab-FOLFOX-4 combination therapy was associated with a 43 % reduction in the risk of progression in patients with *WT KRAS*. Lower risk of disease progression was observed in patients with *WT KRAS*, compared to *MT KRAS* for cetuximab-FOLFOX-4combination therapy. The median PFS of *WT KRAS* with cetuximab alone was about 7.2 months and PFS of *WT KRAS* with cetuximab plus FOLFOX-4 was found to be 7.7 months. For *KRAS* mutation, PFS of cetuximab alone was found to be 8.6 months and cetuximab-FOLFOX-4combination was found to be 5.5 months. The detailed data of quality of life, safety, antibody testing of patients with *WT KRAS* and *MT KRAS* are summarized in Supplementary Table 1A. Data on PFS and OS for *WT* and *MT KRAS* are presented in Supplementary Table 1B.

### Meta-analysis for wild type and mutant type *KRAS*

A meta-analysis was attempted to find out the efficacy of cancer therapy between PFS and OS patients in *WT* and *MT KRAS*. For the analysis, the hazard ratio (HR) and standard error (SE) with 95% CI for antibodies, chemotherapy and combination of both were calculated. The *Q*-test statistic was determined to examine heterogeneity between trials. In addition, the I^2^ value, representing the percentage of total variability attributed to between study heterogeneity was calculated using RevMan 5. We would like to acknowledge the Cochrane community for providing it as free software to perform the forest plot analysis for this study.

A comparative study for PFS and OS of the *WT* and *MT KRAS* colorectal cancer patients has been done with antibody therapy (panitumumab, cetuximab and bevacizumab) alone along with the combination of antibody and chemotherapy (FOLFOX-4).

**Figure 2:**
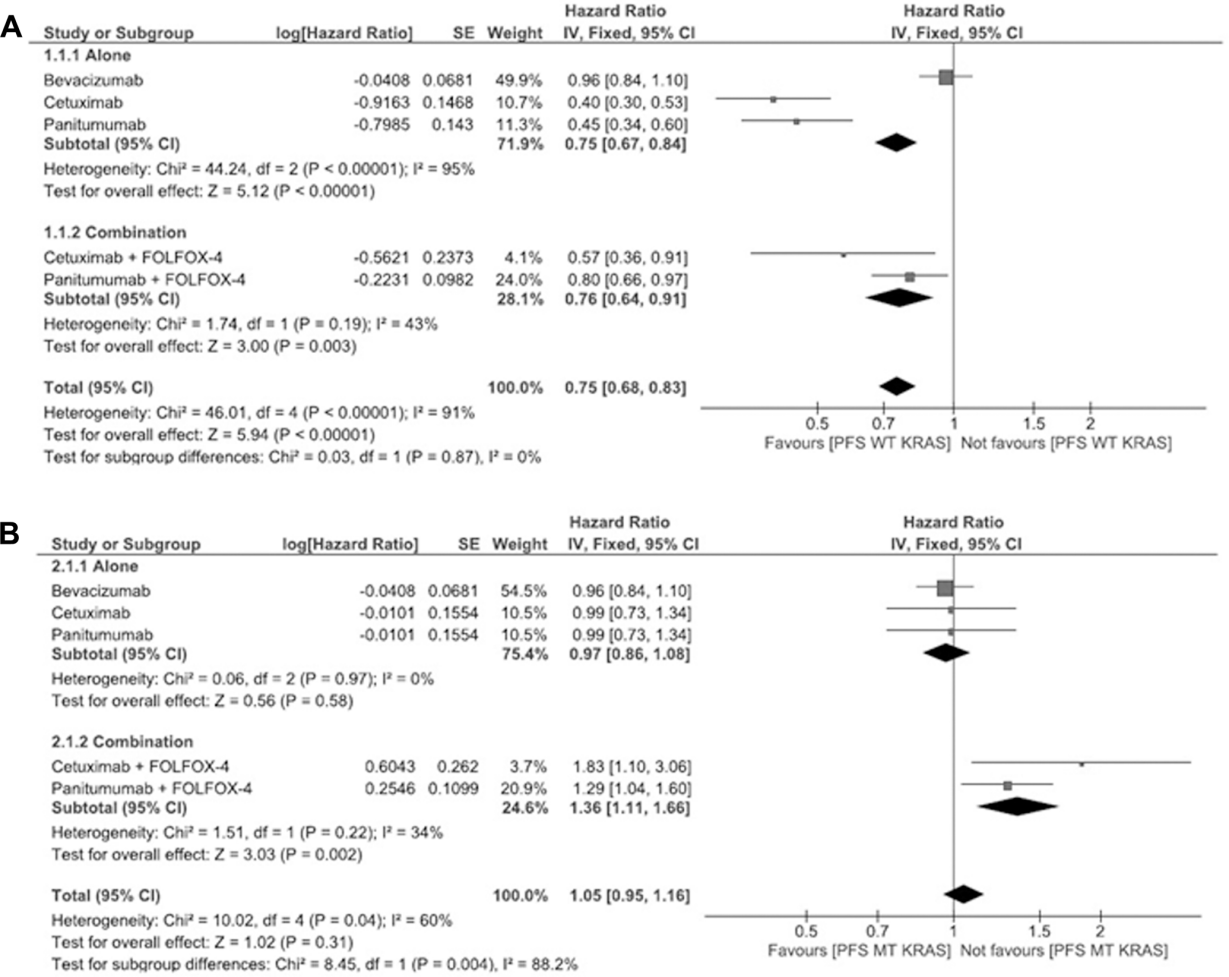
(**A**) Meta-analysis for wild type progression free survival of panitumumab, bevacizumab and cetuximab antibody therapies alone or in combination with FOLFOX-4 (for panitumumab and cetuximab). (**B**) Meta-analysis for mutant type progression free survival of panitumumab, bevacizumab and cetuximab antibody therapies alone or in combination with FOLFOX-4 (for panitumumab and cetuximab).

The PFS for *WT KRAS* is shown in Figure 2A. In this plot, however the sample size seems to be limited; the antibody therapy shows effective treatment when compared to the combination therapy. In this analysis, the total HR for PFS with antibody therapy is 0.75 (95% CI 0.67–0.84), whereas for the combinational therapy, the total HR for PFS being 0.76 (95% CI 0.64–0.91). Thus, the response favors antibody therapy rather than the combination of antibody plus FOLFOX-4 therapy. The PFS for *MT KRAS* is shown in Figure 2B. The result depicts that the combination of antibody plus FOLFOX-4 shows reduced effectiveness, the total HR for PFS being 0.97 (95% CI 0.86–1.08) with antibodies alone and 1.36 (95% CI 1.11–1.66) for combination therapy respectively.

**Figure 3:**
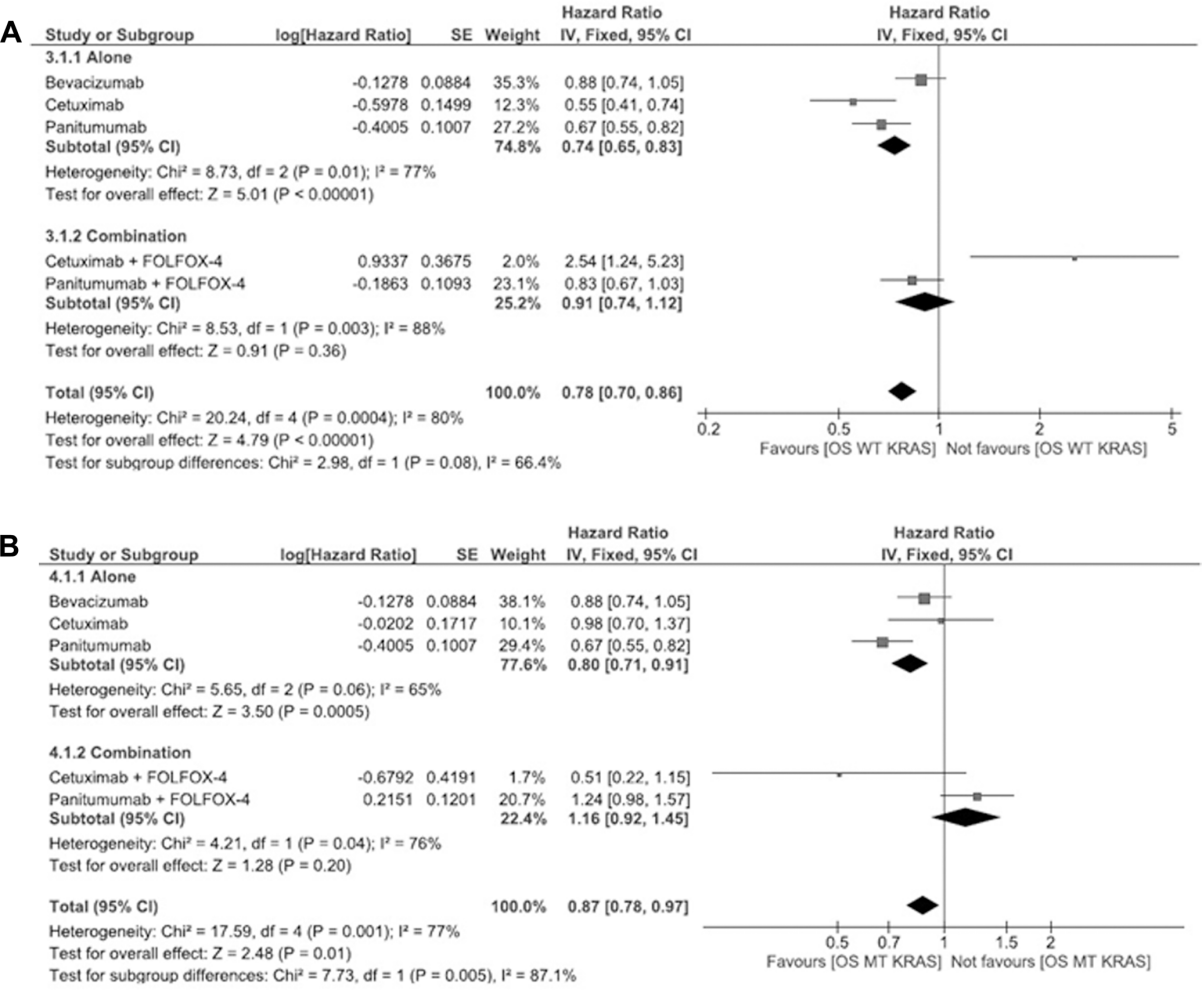
(**A**) Meta-analysis for wild type overall survival of panitumumab, bevacizumab and cetuximab antibody therapies alone or in combination with FOLFOX-4 (for panitumumab and cetuximab). (**B**) Meta-analysis for mutant type overall survival of panitumumab, bevacizumab and cetuximab antibody therapies alone or in combination with FOLFOX-4 (for panitumumab and cetuximab).

The OS for *WT KRAS* is shown in Figure 3A. The pooled HR for OS with antibody therapy in *WT KRAS* patients is 0.74 (95% CI 0.65–0.83) whereas for combinational therapy the pooled HR being 0.91 (95% CI 0.74–1.12). Furthermore, the OS for panitumumab plus FOLFOX-4 patients shows enhanced effect, the pooled HR being 0.83 (95% CI 0.67–1.03).

The OS for *MT KRA*S is shown in Figure 3B. This plot depicts that antibody therapies work better when compared to combination therapy. The pooled HR for OS with antibody therapy in *MT KRAS* patients is 0.80 (95% CI 0.71–0.91) whereas for combinational therapy the pooled HR is 1.16 (95% CI 0.92–1.45).

Furthermore, the OS for panitumumab plus FOLFOX-4 patients shows enhanced effect, for both *WT* and *MT* KRAS when compared to other antibody therapies. The pooled HR for *WT KRAS* is 0.67 (95% CI 0.55–0.82) and for *MT KRAS* is 0.67 (95% CI 0.55–0.82).

From the meta-analysis results (Figures 2A–2B and 3A–3B) it shows that panitumumab and FOLFOX-4 combination is advantageous when considering the *WT KRAS* mutation, but panitumumab alone has enhanced effect when considering the *MT KRAS* patients evaluated for the HR in PFS and OS.

### Cost effectiveness

Analysis of the cost of antibody therapies in combination with chemotherapy indicates that panitumumab-FOLFOX-4 combination represents the most cost-effective therapy compared to bevacizumab, where the minimum expense is ∼40000€. When bevacizumab is used as a first-line therapy for mCRC, it has been equated to an incremental cost-effectiveness ratio (ICER) of more than half a million dollars per quality-adjusted life year. The use of the VEGF inhibitor beyond progression had a cost-effectiveness ratio more than $350,000 whereas the FOLFOX-4 regimen plus cetuximab therapy costs $202484. Thus, panitumumab therapy was found to be cost-effective when compared with its alternatives in the treatment of patients with un-resectable mCRC (Table 4) [18, 19, 20].

**Table 4:** Cost effectiveness for various antibody treatments

ANTIBODY THERAPY+CHEMOTHERAPY	COST EFFECTIVENESS
1. Panitumumab + FOLFOX-4 [18]	Cost starts from 40000€. Panitumumab + FOLFOX added a good value for money.
2. Bevacizumab + FOLFOX-4 [19]	It has been equated to an incremental cost-effectiveness ratio (ICER) of more than half a million dollars per quality-adjusted life year. The use of the VEGF inhibitor beyond progression had a cost-effectiveness ratio of more than $350,000.
3. Cetuximab + FOLFOX-4 [20]	It costs around $202484.

## CONCLUSIONS

Various antibody therapies such as bevacizumab, panitumumab and cetuximab have become available for CRC treatment. These antibody therapies have been used individually or in combination with chemotherapy. This review brings out the most potential antibody therapy in combination with chemotherapy (specifically, FOLFOX-4 has been analysed in the current study). The metaanalysis shows that panitumumab with FOLFOX-4 is advantageous than the other two therapies considering PFS, OS and cost effectiveness as well for the patients with *WT KRAS* mutation. Hence, the results of this study portrays that *KRAS* status might be used as a predictive factor in relation to the efficacy of antibody-chemotherapy and it highlights the increasing importance of tumor biomarker analysis as an element of therapy selection.

## SUPPLEMENTARY MATERIALS TABLES





## Abbreviations

CRC: Colorectal Cancer
mCRC: Metastatic Colorectal Cancer
PFS: Progression Free Survival
OS: Overall Survival
GTPase: Guanosine Triphosphate
IgG_2_: Immunoglobulin G_2_
FDA: Food and Drug Administration
EGFR: Epidermal Growth Factor Receptor
VEGF-A: Vascular Endothelial Growth Factor A
*KRAS*: Kirsten RAS
*WT*: Wild type
*MT*: Mutant type
BSC: Best Supportive Care
ORR: Overall Response Rate
ICER: Incremental Cost Effectiveness Ratio
PRISMA: Preferred Reporting Items for Systematic Reviews and Meta-analyses
HR: Hazard Ratio
SE: Standard Error
CI: Class Interval
