# Supplementary material for: Review on comparative efficacy of bevacizumab, panitumumab and cetuximab antibody therapy with combination of FOLFOX-4 in KRAS-mutated colorectal cancer patients
Source: Oncotarget. 2017 Nov 16;9(7):7739–48. doi: 10.18632/oncotarget.22471 (PMC5800940; doi:10.18632/oncotarget.22471)
Supplement: Supplementary file 1 [file oncotarget-09-7739-s001.pdf]

Review on comparative efficacy of bevacizumab, panitumumab and cetuximab antibody therapy with combination of FOLFOX-4 in *KRAS*-mutated colorectal cancer patients

SUPPLEMENTARY MATERIALS

Supplementary Table 1A: Quality of Life, Safety, Antibody testing of patients. See Supplementary_ Table_1

Supplementary Table 1B: Progression free survival and overall survival

1st Author	Progression Free Survival		Overall Survival	
	**WT KRAS**	**MT KRAS**	**WT KRAS**	**MT KRAS**
Amado et al. [9]	significantly higher *P* < 0.0001<br>0.34 to 0.59 (HR-0.45); 95% CI<br><br>MEDIAN PFS<br>PAN- 12.3 weeks<br>BSC- 7.3 weeks	0.73 to 1.36 (HR-0.99); 95% CI	(Longer survival)<br>0.75 to 1.29 (HR-0.99); 95% CI	0.75 to 1.39 (HR-1.02); 95% CI
Douillard et al. [10]	PAN + FOLFOX-4-199 (61%)<br>FOLFOX-4 alone- 215 (65%)<br><br>PAN+ FOLFOX-4<br>Median PFS, 9.6 (V) 8.0 months<br>0.66 to 0.97 (HR-0.80); 95% CI; *p* = 0.02	PAN + FOLFOX-4- 167(76%)<br>FOLFOX-4 – 157(72%)<br><br>PAN+ FOLFOX-4<br>1.04–1.62 (HR-1.29); 95% CI; *p* = 0.02	PAN+ FOLFOX-4 165 (51%)<br>FOLFOX-4 alone- 190 (57%)<br><br>PAN+ FOLFOX-4<br>Median OS, 23.9 (V) 19.7 months<br>0.67 to 1.02 (HR-0.83); 95% CI; *p* = 0.072	PAN + FOLFOX-4 – 152 (69%)<br>FOLFOX-4 alone- 142 (65%)
Douillard et al. [11]	PAN+ FOLFOX-4 -10.1 months (V) FOLFOX-4 alone-7.9 months<br><br>0.58 to 0.90 (HR-0.72); 95% CI; *p* = 0.004	–	PAN+ FOLFOX-4 (26.0 months)<br>FOLFOX-4 alone (20.2 months)<br><br>0.62-0.99 (HR-0.78); 95% CI; *p* = 0.04	-
Karapetis et al. [13	Median, 3.7 (V) 1.9 months<br>0.30 to 0.54 (HR-0.40); 95% CI; *p* < 0.0001	0.73 to 1.35 (HR- 0.99); 95% CI; *p* = 0.96	1 year survival rate:<br>CTX-28.3%<br>BSC-20.1%<br><br>Median, 3.7 V 1.9 months<br>0.30 to 0.54 (HR-0.40); 95% CI; *p* < 0.0001	CTX- 13.2%<br>BSC- 19.6%<br><br>HR- 0.98; 95% CI; *p* = 0.89
Bokemeyer et al. [14]	179 Patients<br><br>Median PFS<br>FOLFOX-4 alone- 7.2 months<br>CTX+ FOLFOX- 4-7.7 months	FOLFOX-4 alone- 8.6 months<br>CTX+ FOLFOX-4- 5.5 months	FOLFOX-4 alone (HR-0.570); 95% CI; *p* = 0.0163<br><br>CTX + FOLFOX-4<br>Odds ratio= 2.54; *p* = 0.011<br><br>0.358 to 0.907 (HR-0.570); 95% CI.	1.095 to 3.056 (HR-1.830); 95% CI<br><br>At time of analysis,<br>111 patients died<br>CTX+ FOLFOX-4- 60 (35%)<br>FOLFOX-4 alone- 51 (30%)
Sharma et al. [12]	Median PFS 9.3 months<br>7.9 to 10.8; 95% CI; *p* = 0.395	Median PFS 8.7 months<br>5.4 to 15.1; 95% CI	56.60 %<br>*p* = 0.648	50 %

WT KRAS: Wild type K-ras; MT KRAS: Mutant K-ras; CTX: Cetuximab; PAN: Panitumumab; NM-Not Mentioned; HR: Hazard ratio; CI: Confidence interval; BSC: Best Supportive Care; PFS: progression free survival; OS: Overall survival.
