# Supplementary material for: Review on comparative efficacy of bevacizumab, panitumumab and cetuximab antibody therapy with combination of FOLFOX-4 in KRAS-mutated colorectal cancer patients
Source: Oncotarget. 2017 Nov 16;9(7):7739–48. doi: 10.18632/oncotarget.22471 (PMC5800940; doi:10.18632/oncotarget.22471)
Supplement: Supplementary file 2 [file oncotarget-09-7739-s002.docx]

**Table-1A: Quality of Life, Safety, Antibody testing of patients (**WT KRAS: Wild type K-ras; MT KRAS: Mutant K-ras; CTX: Cetuximab; PAN: Panitumumab; - No; NM-Not Mentioned; HR: Hazard ratio; CI: Confidence interval; BSC: Best Supportive Care; AE- Adverse event)

| **1^st^ Author** | Amado et al ^[9]^ | Douillard et al ^[10]^ | | | | | Douillard et al ^[11]^ | Karapetis et al ^[13]^ | | | | | Bokemeyer et al ^[14]^ | | |
| --- | --- | --- | --- | --- | --- | --- | --- | --- | --- | --- | --- | --- | --- | --- | --- |
| **Publication Year** | 2008 | 2010 | | | | | 2013 | 2008 | | | | | 2009 | | |
| **Antibody testing** | NM | Anti-Panitumumab antibodies detected in 3% of 470 patients  Neutralizing antibodies detected in post dose samples in 0.4% of 470 patients | | | | | NM | NM | | | | | NM | | |
| **Quality of Life** | **WT KRAS**  CTX: Improvement in global health status at 8 weeks  BSC: Deterioration in global health status  Difference in CTX & BSC at 8 weeks:  (Mean change in score, −4.7 and −9.6  points, respectively; difference, 4.9; 95% CI, −4.2 to  14.0; P = 0.53) or at 16 weeks (mean change in score,  −9.5 and −13.9 points, respectively; difference, 4.4;  95% CI, −9.2 to 17.9; P = 0.62) | NM | | | | | NM | NM | | | | | NM | | |
| **Safety** | NM | **GRADE 3/4 AE**  Patients with any event  Neutropenia  Skin toxicity  Diarrhea  Neurologic toxicities  Hypokalemia  Fatigue  Mucositis  Hypomagnesemia  Paronychia  Pulmonary embolism  Febrile neutropenia  Infusion-related reaction | **WT KRAS** | | **MT KRAS** | | NM | Patients with any event  Worst grade of 3  Worst grade of 4  Worst grade of 5  Any serious adverse event  Adverse event leading to permanent  Not serious  Serious | **WT KRAS** | | **MT KRAS** | | **MT KRAS:**  **Grade ¾ AE**  Any grade ¾ event  Neutropenia  Rash  Diarrhoea  Leukopenia  Thrombocytopenia  Fatigue  Palmar-plantar Erythrodysesthesia  Peripheral sensory Neuropathy  Anemia  **Composite categories**  Skin reactions  Infusion related reactions | **FOLFOX-4**  117 (70%)  57 (34%)  1 (0.6%)  12 (7%)  10 (6%)  4 (2%)  5(3%)  1 (0.6%)  12 (7%)  4 (2%)  1 (0.6%)  3 (2%)S | **CTX+ FOLFOX-4**  129 (76%)  51(30%)  19 (11%)  14 (8%)  12(7%)  7(4%)  7(4%)  7(4%)  7(4%)  6 (4%)  30(18%)  8(5%) |
|  |  |  | PAN + FOLFOX-4  270 (84%)  136 (42%)  116 (36%)  59 (18%)  52 (16%)  32 (10%)  30 (9%)  28 (9%)  20 (6%)  11 (3%)  9 (3%)  8(2%)  2 (<1%) | FOLFOX-4  227 (69%)  134 (41%)  7 (2%)  29 (9%)  51 (16%)  15 (5%)  10 (3%)  2 (<1%)  1 (<1%)  0  5 (2%)  7 (2%)  - | PAN+  FOLFOX-4  173 (80%)  81 (37%)  66 (30%)  43 (20%)  36 (17%)  19 (9%)  16 (7%)  12 (6%)  13 (6%)  4 (2%)  7 (3%)  7 (3%)  0 | FOLFOX-4  159 (73%)  103 (47%)  3 (1%)  21 (10%)  37 (17%)  8 (4%)  11 (5%)  6 (3%)  1 (<1%)  0  8 (4%)  7 (3%)  - |  |  |  |  |  |  |  |  |  |
|  |  |  |  |  |  |  |  |  | PAN+ FOLFOX4  256 (100%)  146 (57%)  71 (28%)  14 (5%)  110(43%)  65 (25%)  48 (19%)  24 (9%) | FOLFOX-4  248 (99%)  124 (50%)  51 (20%)  16 (6%)  92 (37%)  40 (16%)  28 (11%)  15 (6%) | PAN+ FOLFOX4  266 (99%)  153 (57%)  63 (24%)  19 (7%)  121 (45%)  60 (22%)  50 (19%)  17 (6%) | FOLFOX-4  273 (99%)  146 (53%)  55 (20%)  10 (4%)  84 (31%)  37 (13%)  24 (9%)  14 (5%) |  |  |  |
